# Resin Cement Residue Removal Techniques: In Vitro Analysis of Marginal Defects and Discoloration Intensity Using Micro-CT and Stereomicroscopy

**DOI:** 10.3390/dj10040055

**Published:** 2022-04-01

**Authors:** Mara Gaile, Evaggelia Papia, Vita Zalite, Janis Locs, Una Soboleva

**Affiliations:** 1 Department of Prosthetic Dentistry, Faculty of Dentistry, Riga Stradins University, LV-1007 Riga, Latvia; una.soboleva@rsu.lv; 2 Department of Materials Science and Technology, Faculty of Odontology, Malmö University, 205 06 Malmö, Sweden; evaggelia.papia@mau.se; 3 Rudolfs Cimdins Riga Biomaterials Innovations and Development Centre, Faculty of Materials Science and Applied Chemistry, Institute of General Chemical Engineering, Riga Technical University, LV-1007 Riga, Latvia; vita.zalite@rtu.lv (V.Z.); janis.locs@rtu.lv (J.L.)

**Keywords:** adhesive cementation, discoloration, marginal defect, dental veneers

## Abstract

The objective was to compare marginal defects and evaluate discoloration for adhesively cemented veneers in vitro when using two cement removal techniques. Twenty premolars were prepared with chamfer and borders in enamel. IPS e.max CAD veneers were cemented using Panavia V5 and divided in two groups (*n* = 10): cement excess removed with a probe after tack-curing for 3–5 s, or cement excess removed with a brush, then completely polymerized. All teeth were stored in alginate gel until micro-CT examination. Scanning was performed twice: directly after cementation and after thermocycling (5000 cycles, between 5 and 55 °C). To analyze discoloration, teeth were colored using 0.5% basic fuchsine and examined under a stereomicroscope. Depth of dye infiltration was scored 0 (no discoloration) to 5 (discoloration along the entire margin). Statistically significant differences of cement defects before thermocycling were reported, where brushing showed more defects than probing (*p* = 0.0161). After thermocycling, the defects increased for both groups. Extensive discoloration was the most common (55.56%) when removing excess by probing; by brushing, 90% of the specimens exhibited slight discoloration (*p* = 0.008). Regression analysis showed no relationship between type of defect and degree of discoloration. Removing cement with a brush causes more marginal defects, however less discoloration after thermocycling.

## 1. Introduction

Adhesively cemented porcelain veneers are widely used in aesthetic dentistry, and the clinical outcome has been proven to be with good prognosis, especially if the preparation is located completely in enamel and if correct adhesive treatment procedures are carried out [1]. In the literature, it is reported that porcelain veneers show high long-term survival rates, such as 87–94% after 9 years, depending on the used material [2]. Data from a recent systematic literature review with an emphasis on complications reports 95.5% in a 10-year estimated cumulative survival rate (CSR) with veneer fracture, debonding, secondary caries, and need of endodontic treatment being the most common reasons for failure. Fracture and debonding were more commonly happening within the first years after cementation [3]. Another systematic review with meta-analysis cites severe marginal discoloration (2%) as one of the frequent issues, apart from those mentioned previously [2].

The use of adhesive cementation results in significant strengthening of the ceramic restoration, thereby impacting the clinical performance. The strengthening is dependent on the creation of a resin–ceramic hybrid layer sensitive to cementation variables such as ceramic surface treatment and the type of resin-based cement, as well as clinical handling technique [4].

Hydrofluoric acid etching of ceramics provides surface roughening, which significantly increases the bond strength. Etching is considered the most significant factor in creating high bond strengths between ceramics and resin-based cements [5,6,7]. An etched ceramic surface is coated with silane, which acts as a coupling agent and improves adhesion with resin cements [8]. Silanes are bifunctional molecules that bond silicone dioxide with the OH groups on the ceramic surface. They also have a degradable functional group that copolymerizes with the organic matrix of the resin. The application of a silane coupling agent to the etched ceramic surface provides a chemical covalent and hydrogen bond [9].

Different resin cement systems have been suggested for the cementation of porcelain veneers. There are several factors that have to be considered when choosing the most appropriate cement for each clinical situation, such as the properties of the restorative material used, working times, cost, technique sensitivity, as well as the possibility to clean cement residue. Various cement removal and finishing techniques are recommended by different authors; for example, removing unpolymerized residual cement with a brush moistened with a bonding agent [10,11], tack-curing (initial short light curing to achieve a gel-like state for easier excess cement clean-up) [12], or combining multiple techniques (removing unpolymerized cement excess with a brush, followed by tack-curing and removal with a probe) [13], which could influence long term results of cementation [10,12,13]. Since possible color change (discoloration of the margin) is a very common problem for porcelain veneers in the long term, it is important to understand the most optimal procedure to minimize it. Thus far, possible effects of various cement removal techniques on discoloration have not been widely investigated. Therefore, in the current study, two techniques suggested by the manufacturer were compared: tack-curing following removal with a probe, or removal with a brush before complete polymerization. The hypothesis of the study was that the number of defects in the cement layer correlate with discoloration intensity.

The aim of this study was to compare two different resin cement removal techniques by evaluating the number of marginal defects and levels of discoloration in vitro.

## 2. Materials and Methods

### 2.1. Inclusion of Samples

This in vitro study included 20 intact premolars extracted at the RSU Department of Oral and Maxillofacial surgery (April 2020 to October 2020) due to orthodontic or periodontal indications. The study was approved by the local research and ethics committee (permit number 6-3/5/46). Exclusion criteria were as follows: caries, extensive restorations, abrasions, cracks, and hypocalcifications. Teeth were manually cleaned of plaque, calculus, and remaining periodontal tissue using an ultrasonic scaler (PiezoLED, KaVo Dental) and kept in physiologic saline solution in a refrigerator until the beginning of the study. All teeth were tested less than 6 months after extraction. Specimen preparation, cementation, and measurement principles were developed together with a supervisor and all following steps were carried out by a single operator.

### 2.2. Tooth Preparation

**Figure 1:**
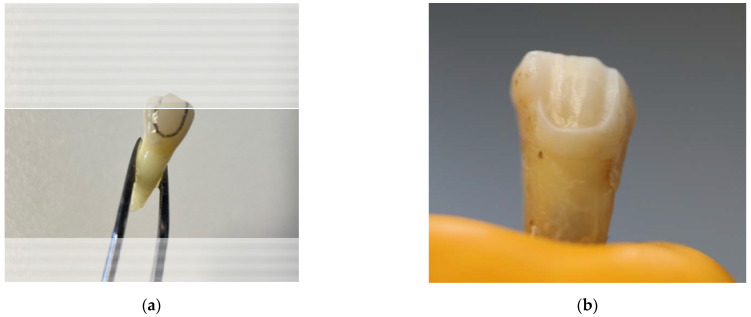
Teeth preparation steps: (**a**) preparation outline marked on the tooth surface; (**b**) tooth after preparation.

The shape of each tooth preparation was prefabricated from adhesive tape to ensure similar dimensions for all preparations. The preparation outline shape was placed on the vestibular surface of each tooth and marked with a waterproof marker before starting the preparation. Preparations were undertaken using a cylindrical round end diamond bur. Vestibular surfaces of teeth were prepared approximately 0.5 mm deep with rounded corners and a chamfer finish line, keeping enamel around all borders (Figure 1a,b).

### 2.3. Veneer Fabrication

**Figure 2:**
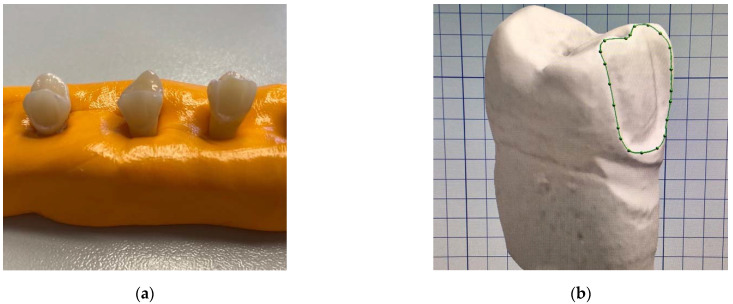
Veneer fabrication: (**a**) teeth fixed in putty silicone bases to ensure better control during scanning and cementation; (**b**) design of the veneers with the margin line marked (green line).

After preparation, the teeth were fixed in putty silicone bases to ensure better control during further clinical and laboratory steps (Figure 2a). The prepared teeth were scanned using a Ceramill Map 600 scanner (Amann Girrbach AG, Koblach, Austria), and the designing of each veneer was undertaken using Ceramill Mind software (Amann Girrbach AG) (Figure 2b). The cement gap was set to 0.02 mm, the milling device was a Ceramill Motion 2 (Amann Girrbach AG), and IPS e.max CAD (Ivoclar Vivadent, Zurich, Switzerland) material was used for milling the veneers. All further laboratory steps were performed according to manufacturer instructions for use, using crystallization and firing parameters provided by the manufacturer [14].

### 2.4. Cementation

**Figure 3:**
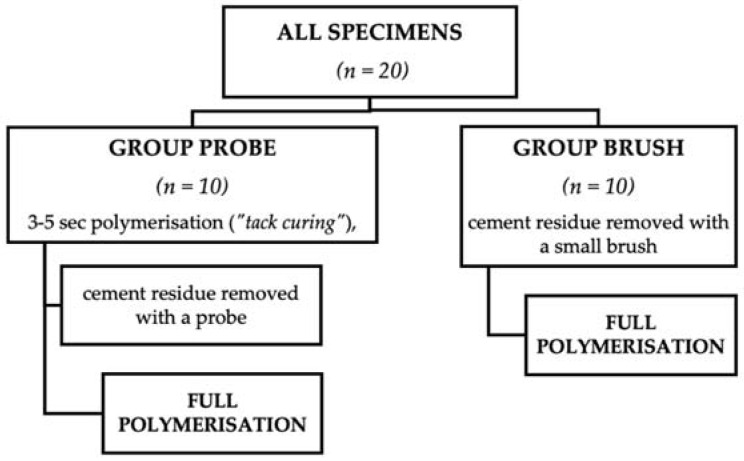
The study groups with the cementation and two different cement removal techniques.

All specimens (*n = 20*) were divided in two groups before cementation, as shown in Figure 3.

The Panavia V5 (Kuraray Noritake, Tokyo, Japan) cement system was used for cementation. Cementation steps were carried out by the same operator according to the instruction provided by the cement manufacturer [15], and two cement removal techniques recommended in the instruction were used: tack-curing and following removal with a probe, or removal of unpolymerized cement excess with a brush. Before cementation, the teeth were fixed in putty silicone blocks to ensure stabilization and the veneers were cemented, adapting them on a tooth surface with an instrument and then using finger pressure. After cementation, restoration margins were thoroughly polished using polishing discs (Sof-Lex, 3M) and rubber polishers. After the cementation procedure, each specimen was placed in a plastic container (40 mm length; 10 mm diameter) in translucent alginate gel and stored in a room temperature of approximately 20 °C before further examination.

### 2.5. Thermocycling

Thermocycling (Thermocycler 1100/1200, SD Mechatronik, Feldkirchen-Westerham, Germany) was performed for 5000 cycles. A cycle lasted for a total of 60 s: 20 s in two baths containing distilled water, with temperatures of 5 and 55 °C, respectively, and with 10 s transfer time between the baths.

### 2.6. Micro-Computed Tomography (Micro-CT) Evaluation

**Figure 4:**
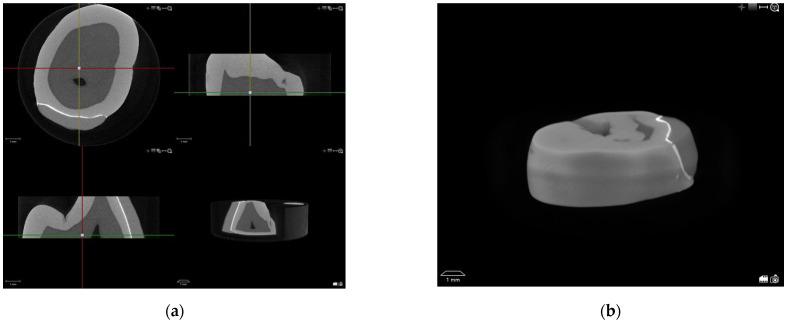
Process of setting the intervals for scanning: (**a**) positioning of the specimen and measurements; (**b**) segments chosen for micro-CT scanning.

All specimens (*n* = 20) were examined using micro-CT (µCT 50, Scanco Medical AG, Wangen, Switzerland) to assess the amount and character of marginal defects. Alginate gel-filled containers with the specimens were fixed in sample holders that were rotated to provide adequate X-ray exposure. Parameters used for scanning are shown in Table 1. Scanning was conducted in a segment, starting 2 mm from the tip of the cusp (Figure 4), with the intent to exclude any anatomical differences. For each tooth, 413 micro-CT slices were obtained.

After scanning, both sides of each specimen were examined, and data were collected using MS Excel.

Before and after thermocycling for each specimen, micro-CT images were evaluated:

1. Number of structural defects next to enamel, next to the veneer, and in the cement layer;
2. Sum of overall structural defects;
3. Number of slices with cement layer underfill/irrelevant overfill/wide overfill.

**Figure 5:**
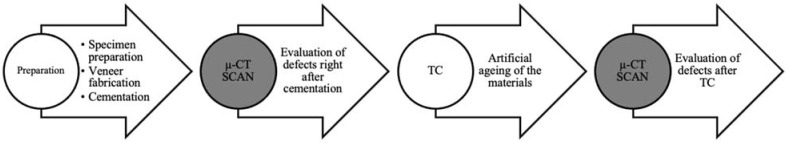
Data collection steps. TC = thermocycling.

Micro-CT examination was conducted twice. Data collection steps are shown in Figure 5.

### 2.7. Coloring

**Figure 6:**
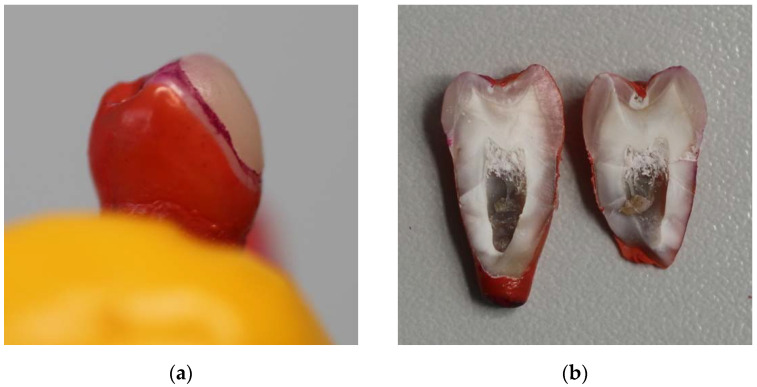
Specimen preparation and coloring: (**a**) teeth were immersed in 0.5% basic fuchsin dye for 24 h. After coloring, teeth were rinsed thoroughly under running water and polished with a polishing brush to remove the dye on the surface; (**b**) each specimen was sectioned at the center in facio-lingual direction with a 0.5 mm low speed diamond disk.

After thermocycling, coloring of the specimens was performed according to the method proposed in the previous study by Haralur et al. (2018) [16]. Apical foramina were closed with glass ionomer cement before coloring. All tooth surfaces, except for 1 mm around the veneer margin, were coated with nail varnish and then immersed in 0.5% basic fuchsin dye for 24 h. After coloring, the specimens were polished thoroughly to remove the dye on the surface and sectioned facio-lingually using a 0.5 mm low speed diamond disk (Figure 6). One veneer debonded during the sectioning process, and thus was excluded from further microscopic examination.

Both hemi-sections were evaluated under a stereomicroscope (Leica MZ16 A, Leica Microsystems, Wetzlar, Germany) using 20× magnification. All micro-CT measurements and microscopic evaluation of discoloration were undertaken by one calibrated operator.

**Figure 7:**
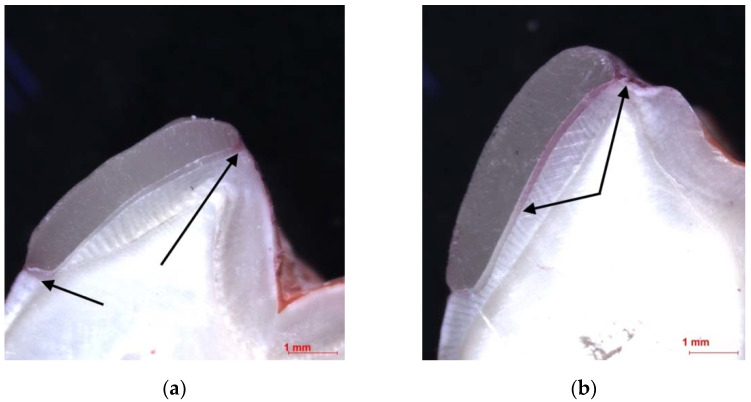
Stereo microphotography showing different discoloration intensities: (**a**) slight cement layer discoloration, 20× magnification; (**b**) extensive cement layer discoloration, 20× magnification.

Intensity of discoloration was evaluated according to the scheme: 0—no discoloration; 1—slight discoloration (not exceeding chamfer preparation margins cervically/edge occlusally); 2—extensive discoloration (color diffused deeper in the cement layer) (Figure 7).

Additionally, the depth of dye penetration was scored 0 to 5 according to the scheme proposed previously by Haralur et al. (2018) [16].

### 2.8. Statistical Analysis

Specimen groups were encrypted for the statistical analysis. Mean values and standard deviations were calculated for the type of defect and degree of discoloration (0–5). Statistical significance of differences in mean values between groups before and after thermocycling were assessed using Mann–Whitney and Wilcoxon signed rank tests. Difference in distribution of discoloration between groups was assessed using the Fischer exact test. Multivariate regression analysis (mvreg) was performed to assess a possible relationship between the type of defect and degree of discoloration. The level of significance was set to α = 0.05.

## 3. Results

### 3.1. Size and Character of Defects

**Figure 8:**
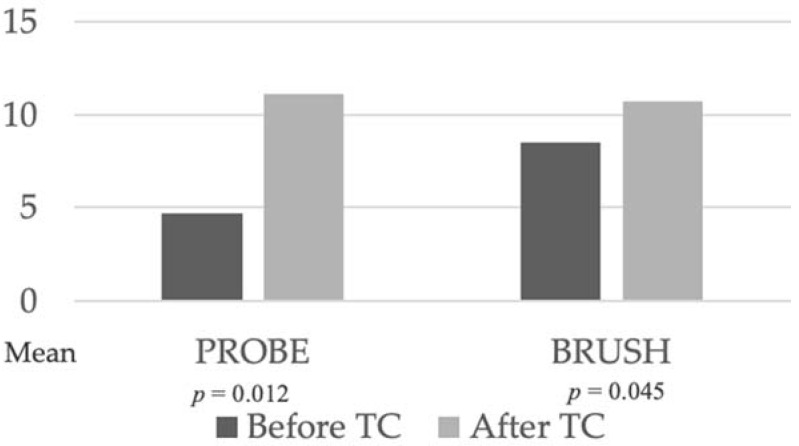
Comparison of structural cement layer defects between groups before and after thermocycling.

There were statistically significant differences in mean value of structural cement layer defects between probe group and brush group before thermocycling. Before thermocycling, brushing showed more defects in total than the probe group (*p* = 0.0161). After thermocycling, total count of defects increased for both groups (Figure 8).

**Figure 9:**
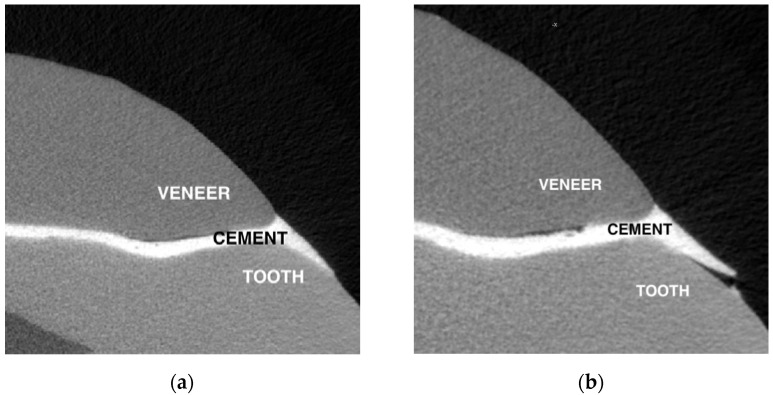
Sample number 13 before and after thermocycling: (**a**) small defects and cement layer overfill before thermocycling; (**b**) after thermocycling, defects adjacent to enamel and veneer are visible.

Example of defect progression on micro-CT scans after thermocycling is visible in Figure 9, where small defects and cement layer overfill are visible.

The brush group had a higher mean value of defects in the cement layer prior to thermocycling than the probe group (*p* = 0.0225), which showed more wide overfills (*p* = 0.0476). For detailed distribution of defect types and progression in both groups, see Table 2.

### 3.2. Comparison of Discoloration

#### 3.2.1. Intensity of Discoloration

**Figure 10:**
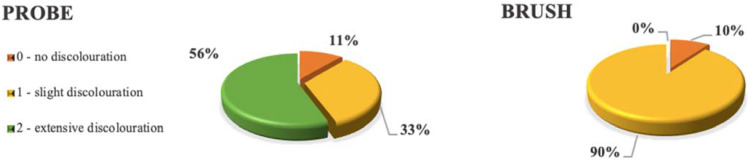
Comparison of discoloration presence between two groups.

Only one specimen from each group had no discoloration (score 0). In the probe group, one-third of the specimens exhibited slight discoloration (score 1), and extensive discoloration (score 2) was the most common in this group (55.56%). In the brush group, 90% of the specimens exhibited slight discoloration (score 1). Extensive discoloration was not observed in the brush group at all (*p* = 0.008) (Figure 10).

#### 3.2.2. Depth of Dye Penetration

Analysis of the dye penetration depth showed a higher mean for the probe group (2.67 SD = 2.00 vs. 1.10 SD = 0.57; *p* < 0.001).

Multivariate regression analysis showed no relationship between the type of defect and degree of discoloration.

## 4. Discussion

The results of our study show that the number of defects does not correlate with discoloration intensity, although initially it was expected to have a connection between a higher degree of discoloration and increased number of defects. An incomplete polymerization could be one of the factors influencing discoloration intensity. The effect of different polymerization strategies should be investigated further since the group where tack-curing was undertaken showed more discoloration, despite having less cement layer defects. There is a remaining question about the influence of continuous polymerization in preventing cement layer discoloration since the group where tack-curing was performed showed more discoloration, despite having less cement layer defects.

Clinical success depends on degree of conversion (DC), which influences polymerization shrinkage (PS) of resin cement. Dual-cure resin cements can either be light cured or used in self-cure mode since they contain both photo and chemical initiators. However, light curing of these cements is beneficial to achieve optimal DC and minimize PS [17]. As cements with high shrinkage are more dimensionally unstable, they can lead to marginal leakage and discoloration [18]. Few studies on tack-curing have been undertaken previously. Further, it has been concluded that tack-curing causes slight surface hardness reduction and slightly lower DC in resin cements, but this should not be significant clinically [12,19]. However, aforementioned are in vitro studies with no clinical or long-term evaluation, and additional research on this topic would be useful since our study shows that the tack-cured group had more discoloration after thermocycling, despite having less cement layer defects.

Color stability and resistance to staining are important clinical aspects when choosing appropriate cement for veneer cementation. The literature reports that dual-polymerization and light-polymerizing resin-based cements both show good clinical performance when used for cementation of porcelain veneers [20], and they show similar color stability [21,22]. However, marginal discoloration increases over a 2-year period for both cement systems [22]. Resin cements tend to show varying degrees of discoloration, especially at the exposed cement margins for dual-cure cements [23]. Apart from curing mode, there is also a discussion about the most appropriate pre-treatment protocol and adhesive. Adhesives have evolved tremendously over the years and have been made more simple for use. However, simplicity does not always mean improvement, and it is very important to choose the most appropriate adhesive for each clinical situation and to follow the manufacturer’s instructions precisely [24]. Self-adhesive self-cure cement systems show inferior color stability when compared to that of the total-etch light-cure cement or self-adhesive dual-cure cement [25]. In an article by Haralur et al., where different pre-treatment methods and cement systems were compared, the etch-wash method with a dual-cure cement system showed the lowest interfacial microleakage in cervical and incisal regions when compared to etch-wash light cure, self-etch, and self-adhesive techniques. The highest leakage was recorded with self-adhesive resin luting cements [16]. These findings were confirmed in another study, where the results were explained by an insufficient etching ability of the cement to smear layer-covered enamel. Therefore, phosphoric acid etching is recommended prior to the self-etch adhesive application to adequately etch enamel before cementation of porcelain veneers [26]. Another additional pre-treatment step, especially when bonding to dentine, is surface sandblasting prior to etching, which has shown to increase adhesive strength [27].

Universal adhesives that can be used for direct and indirect restorations have gained popularity due to the simplicity of use and their effectiveness (can bond to methacrylate-based restoratives and cements, tooth, metal, zirconia), thanks to the inclusion of 10-methacryloyloxydecyl-dihydrogen-phosphate (10-MDP) [24]. Based on available information from the literature in the current study, the 10-MDP-containing dual-cure resin cement system Panavia V5 was used because it displays low technique sensitivity and provides good adhesion to enamel and to dentin [28]. It has been shown that Panavia V5 can be successfully combined with different CAD/CAM (computer-aided design/computer-aided manufacturing) ceramic materials in vitro (including IPS e.max CAD used in this study); these combinations are also suggested for clinical application [29]. In addition, Panavia V5 shows the highest shear bond strength—specifically between IPS e.max CAD ceramics and dentin—when compared to self-adhesive, self-etching cements [30], as well as the lowest degree of microleakage in comparison to other dual-cure cements [31].

In the current in vitro study, intact premolar teeth that were extracted due to orthodontic or periodontal indications were used. Premolar location is not the most typical for porcelain veneers, and experiment results for incisor group teeth could be slightly different because of discrepancies in tooth size and anatomy. However, these differences may not be clinically significant since one of the main factors influencing outcome is the presence of enamel around all borders [1]. Another potential limitation of the study is the relatively small sample size confined to our inclusion/exclusion criteria and limited availability of biological material.

There is an ongoing discussion about whether machinable or heat-pressed veneers provide better adaptation. In an in vitro study from 2012, pressable ceramic laminate veneers showed better marginal adaptation, thinner cement film thickness, and improved resistance to microleakage when compared to machinable ceramic veneers [32]. More recently, a clinical trial with a 2-year follow-up reports that the fabrication method, whether CAD/CAM or heat-pressed, has no effect on the marginal and internal adaptation of porcelain laminate veneers, and both techniques perform well clinically [33]. Since CAD/CAM techniques are constantly developing and are used more and more frequently in clinical settings, the decision was made to use the digital manufacturing process in the current study.

In the current study, specimens were fixed in putty silicone blocks to ensure stabilization and veneers were cemented, adapting them on a tooth surface with an instrument and then using finger pressure. Other techniques, such as the use of ultrasound, have been reported in previous in vitro studies as being successful in reducing the gap distance at the tooth/ceramic interface [34]. Moreover, constant seating pressure during the adhesive cementation procedure (1.25 MPa for 3 min) has the potential to improve microtensile bond strength [35]. This variable was not monitored in this study and could have influence on the width of cement gap between the tooth and the veneer. However, this is applicable to clinical conditions where seating pressure is also not monitored routinely.

During the veneer cementation process, it is critical to ensure that all border irregularities are filled with resin cement, which is polished well after polymerization. However, non-polished cement overfill is frequently seen around veneer borders [36]. The present study reports similar results, with different degrees of cement overfills present in both groups. These findings can be easily explained since non-polished cement overfills were visible well under microscope and in micro-CT scans under great magnifications. However, clinically it would not be possible to examine the borders of each veneer under such circumstances. Interestingly, overfills decreased after thermocycling, which could be explained by composite resin matrix dissolution in water following cement contour smoothening. The question remains—How clinically significant are such small amounts of cement overfill, and can they cause any problems in the long term, such as cement layer discoloration?

One of the factors that influences the outcome of an in vitro study conducted on extracted teeth is specimen storage and sterilization. When comparing different types of storage media, it was shown that saline and 5.25% NaClO significantly lowered shear bond strength values (composite to dentin) and are not recommended for such studies. Sterilization with an autoclave of teeth initially stored in saline and formalin also had negative effects in contrast to only formalin storage [37]. Dry specimen storage following rehydration (in distilled water 2 weeks prior to use) is also a possibility for in vitro studies, although it was not usable in this case since it has a negative side effect of crack formation through the dry enamel and occasionally separation of enamel from dentine [38]. In the current study, to avoid the possibility of drying, all teeth were kept in separate containers in alginate gel and only removed during preparations, scanning, and cementation, as well as the thermocycling process, since it is recommended to keep specimens hydrated during the entire experimental process. Additionally, post-extraction time did not exceed 6 months, as recommended for in vitro testing [39]. Alginate gel-filled containers were used to fix specimens in sample holders during micro-CT scanning to avoid artifacts caused by specimen movements. Alginate gel has great potential as a biomaterial for various biomedical applications as it is easy to use, biocompatible, allows the maintenance of a physiologically moist environment, and can be simply modified to prepare alginate derivatives with new properties [40]. It was chosen as the appropriate media for the study also due to the fact that it is radiolucent, and thus not interfering with the scanning process.

Thermocycling was used to simulate aging of dental materials. The aging process influences resin cement, and therefore can affect the color of porcelain veneers [41] and their marginal adaptation, since it is known that the dissolution of composite resin matrix happens in oral liquids [42,43,44]. For in vitro studies to artificially age dental materials, different methods, for example, water storage or thermocycling, are used. Thermocycling between 5 and 55 °C simulates the effect of varying temperatures present in the oral cavity due to hot and cold foods and drinks [45,46]. Although most authors are in agreement about the temperatures, there is a great diversity of opinions regarding the number of cycles. In the current study, thermocycling was performed for 5000 cycles, with temperatures between 5 and 55 °C. In other studies, suggested duration of thermal cycling varies, starting at 100 cycles [47] to 3000 and more [48,49,50], and reaching up to 100,000 cycles [51]. However, beyond 5000 cycles the flexural strength of the composite materials hardly changed [52]. According to the International Association for Standardization (International Standards Organization, 1994. Guidance on Testing of Adhesion to Tooth Structure. ISO/TR 11405, Dental Materials), a protocol of 500 cycles is appropriate in simulating the aging of biomaterials.

There is no consensus about the number of thermal cycles corresponding to usage time in vivo, and suggestions for a specific number of cycles are not based on researched data. Although thermal cycling is one of the most widely used testing methods for dental materials, there is a lack of a standardized protocol, which makes it hard to compare the study results [53]. In the context of material aging, it should be noted that a major limitation of in vitro studies is not taking into account additional factors influencing individual treatment outcomes, such as oral pH value, composition of saliva, and food and oral hygiene regimens, which vary from patient to patient.

The micro-CT method can be successfully applied to investigate mineralized tissues, such as teeth and bone, and different materials, such as ceramics and polymers. It was used as a method of choice in the present study. Although micro-CT has been widely used in dentistry to study various topics in endodontics and orthodontics, there is a limited number of studies regarding prosthodontic questions. Only a few authors have used micro-CT to investigate topics such as biomechanics of different veneer preparation designs [54], cement thickness and stress distribution in ceramic crowns [55], and cement layer around root canal posts [56,57,58]. Interfacial gap has been studied using micro-CT in regard to class II cavities [59] and in a few studies regarding marginal gap and cement layer specifically around veneers [60,61,62]. The micro-CT method is easy to use and gives a good insight into material structure, behavior, and precision, as well as defects. Therefore, it is a good fit for analyzing prosthetic restorations, which often include combinations of multiple material layers. It is debatable though if all the defects visible in a micro-CT scan are relevant clinically since our specimens showed no correlation between the number of defects and intensity of discoloration. Another limitation is possible measurement errors. In the current study, scanning was conducted in an interval, starting 2 mm from the tip of the cusp for all teeth to exclude any anatomical differences. Although all micro-CT scans were undertaken in a similar manner, minor shifts in scanned segments cannot be excluded, which could lead to some degree of measurement errors when comparing scans before and after thermocycling. To minimize the possibility of measurement mistakes, all micro-CT measurements and microscopic evaluation were performed by one calibrated operator. However, it has to be noted that the evaluation of defects and discoloration is subjective.

One of the limitations is not considering possible differences in marginal and cervical regions of the veneer, since all scanned segments were located in the middle portion of a tooth. A small percentage of veneers show immaculate margin adaptation [63]; and, although veneer manufacturing techniques have evolved, in recent studies it is shown that cervical portion can still be more challenging in terms of adaptation [64]. It also has to be noted that polishing instruments work well on flat, easily accessible surfaces. However, it is complicated to ensure the same quality around interproximal areas and gingival margin [1]. The microleakage at the cervical tooth–composite interface is also shown to be higher in comparison to the incisal interface [16]. 

## 5. Conclusions

Removing unpolymerized cement with a brush caused more defects in the cement layer when compared to tack-curing and removing excess cement with a probe. Cement removal with a brush before light curing showed less discoloration after thermocycling. The type of defect did not have a correlation with discoloration.

Further research is required to understand the connection between the type of defect and depth of discoloration in order to determine the best cement removal method for improving cementation quality for porcelain laminate veneers

## Figures and Tables

**Table 1:** Parameters used for scanning.

Energy	90 kVp
Intensity	66 µA
Filter	Al 0.5 mm
Resolution	High
Voxel size	7.4 µm
Integration time	1000 ms
Data Average	3

**Table 2:** Localization and character of defects in two groups before and after thermocycling.

Type of Defect	PROBE					BRUSH				
	Before TC		After TC		*p* Value	Before TC		After TC		*p* Value
	Mean	SD	Mean	SD		Mean	SD	Mean	SD	
Overall count	4.7	1.83	11.1	5.99	**0.0123**	8.5	4.22	10.7	3.89	**0.0453**
In cement layer	3.7	2.21	6.7	3.83	**0.0151**	6.9	3.48	6.5	2.37	0.8768
Next to tooth	0.7	0.82	2.8	1.62	**0.0167**	0.7	0.82	1.7	1.57	**0.0165**
Next to veneer	0.3	0.95	1.6	1.84	0.0892	0.9	1.37	2.5	1.51	**0.0327**
Underfill	112.9	174.79	99	86.22	0.6080	210.6	159.31	150.1	175.75	0.1610
Wide overfill	201.9	170.55	150.7	129.29	**0.0474**	55.9	79.29	55.2	78.99	0.1579
Irrelevant overfill	373.8	110.06	299.2	103.89	**0.0469**	389.4	185.06	314.3	176.15	**0.0050**

## Data Availability

The data that support the findings of this study are available from the corresponding author, M. Gaile, upon request.
